# Illinois Dental Directory

**Published:** 1910-03-15

**Authors:** 


### BIBLIOGRAPHY.

Illinois Dental Directory. The Illinois Dental Society has just published a complete directory of the profession in that State. It has several unique and admirable features. It is possible to locate for instance the dentists in any district of the city of Chicago, which is a very great convenience. The book contains much valuable information. Such as a history of the State Dental Society and its present organization; The new Code of Ethics; The Illinois Law; List of the Officers of all the State and component societies; maps showing geographical location of all component societies, and much other valuable information. The book shows the handiwork of Dr. Arthur Black, and like all such things done by him leaves little to be desired. It may be bought for two dollars.

